# The Effects of Irreversible Electroporation on the Colon in a Porcine Model

**DOI:** 10.1371/journal.pone.0167275

**Published:** 2016-12-01

**Authors:** Xiaomei Luo, Xianjun Liang, Jiannan Li, Jian Shi, Wenlong Zhang, Wei Chai, Jiuping Wu, Shuai Guo, Gang Fang, Xulong Zhou, Jianhua Zhang, Kecheng Xu, Jianying Zeng, Lizhi Niu

**Affiliations:** 1 School of Medicine, Jinan University, Guangdong Province, Guangzhou, China; 2 Department of Gastrointestinal Surgery, Taizhou Central Hospital of Zhejiang Province, Taizhou, China; 3 Department of General Surgery, The Second Hospital of Jilin University, Changchun, China; 4 Department of Hematology and Oncology, China-Japan Union Hospital of Jilin University, Changchun, China; 5 Department of Gynecology and Obstetrics, The First Hospital of Jilin University, Changchun, China; 6 Department of Spinal Surgery, Orthopaedic Hospital of the Second Hospital of Jilin University, Changchun, China; 7 Department of Endocrinology, Taizhou Central Hospital of Zhejiang Province, Taizhou, China; 8 Department of Surgery and Anesthesia, Fuda Cancer Hospital, Jinan University School of Medicine (Guangzhou Fuda Cancer Hospital), Guangzhou, China; 9 Department of Pathology, Fuda Cancer Hospital, Jinan University School of Medicine (Guangzhou Fuda Cancer Hospital), Guangzhou, China; 10 Department of Endoscopy, Fuda Cancer Hospital, Jinan University School of Medicine (Guangzhou Fuda Cancer Hospital), Guangzhou, China; 11 Guangzhou Fuda Cancer Institute, Fuda Cancer Hospital, Jinan University School of Medicine (Guangzhou Fuda Cancer Hospital), Guangzhou, China; Consiglio Nazionale delle Ricerche, ITALY

## Abstract

**Background and Aim:**

Irreversible electroporation (IRE) is a method of targeted cell ablation which has been suggested as a potential cancer therapy as it leaves structures such as blood vessels and the extracellular matrix intact, thereby allowing the rapid recovery of healthy tissue. Here, we investigated the effects of IRE on the colon *in vivo* in a porcine model.

**Methods:**

IRE ablation was performed on the colon walls of 12 female Tibet mini-pigs, creating a total of 24 lesions. Lesions were monitored periodically by endoscopy. The pigs were euthanized 7, 14, 21 or 28 days after IRE ablation and the colons harvested for gross and histological analysis. Sections were stained with hematoxylin and eosin (H&E), Masson’s trichrome (MT) stain and terminal deoxynucleotidyl transferase dUTP nick end labeling (TUNEL) assay.

**Results:**

All pigs tolerated the ablation procedure without serious clinical symptoms or complications. There was no evidence of perforation by endoscopy or gross postmortem examination. All lesions were characterized by necrotic cell death with mild inflammation and hyperemia, with a sharp demarcation between ablated and adjacent normal tissue. A fibrous scar was observed in the ablated colon tissue. Histological analysis revealed damage to each layer of the colon. Histopathology findings also showed the preservation of extracellular structures and the recovery of the ablated colon.

**Conclusions:**

The complete ablation of the target area, its rapid recovery and the lack of posttreatment symptoms suggest that IRE ablation may be a promising therapy for tumors located adjacent to or violating the colon wall.

## Introduction

Colon cancer is one of the most common malignant tumors in the world, accounting for approximately 1.2 million incident cases and over 600,000 deaths annually[1]. The five-year survival rate for patients with locoregional disease is approximately 70% but decreases to 13% after the cancer has spread to distant organs[2]. Although the overall outcomes remain poor because of disease progression, many patients nevertheless retain a good performance status and could benefit from additional treatment options[3, 4]. Collateral damage to the colon often occurs after radiotherapy for pelvic or abdominal malignancies or as a side effect of chemotherapy, resulting in bloating, abdominal cramping, severe diarrhea, nausea, and vomiting. These side effects are seen as the limiting factor in increasing both chemotherapy and radiotherapy dosage and can force discontinuation of treatment[5]. Irreversible electroporation (IRE), a non-thermal ablation modality, has recently been shown to be a precise and complete method of tumor ablation. IRE utilizes microsecond pulses of electric field to produce permanent nanopores in the cell membrane and results in selective cell death while preserving all other molecules, including the structural integrity of blood vessels, nerves, and extracellular matrix. In the treatment of abdominal cancer, IRE has the ability to avoid collateral damage even in tissues within the electric field[5, 6]. This method of targeted induction of cell death has proved effective in destroying cancer in animal models[7–12].

A number of previous studies have investigated the effect of IRE on the small intestine and rectum. Onik et al. reported that there was no effect of IRE on the rectum in a canine model[10]. Srimathveeravalli et al. found that an endorectal electrode can be used to deliver IRE and create limited focal ablations in the rectal wall, and that treatment parameters can be determined through numeric modeling to control the depth of penetration of ablation[13]. Schoellnast et al. found that IRE ablation adjacent to the rectum may be uneventful if the rectum wall is mobile and able to contract. Chronic inflammation and flbrosis of the muscularis propria were essentially limited to the external tissue layer[14]. Phillips et al. found that when IRE ablation was performed on the small intestine it led to complete cell ablation in the target tissue without any physiological effects [7]. IRE may therefore be advantageous in patients with tumors that may be adjacent to the rectum or other bowel, such as carcinomas of the bladder, colon, reproductive organs, and pancreatic cancer[10].

Up to now, there has been no systematic study on how IRE affects colon tissues and the process of tissue regeneration. Therefore, the purpose of our research was to study the effect of IRE ablation on the colon tissue and evaluate the safety of IRE ablation on the colon wall in a porcine model. Our hypothesis was that the colon would remain structurally intact after IRE ablation, survive the treatment, and recover due to the ability of IRE to spare the extracellular matrix.

## Material and Methods

### Experimental animals

The study was approved by the Research Animal Care and Use Committee of Guangzhou Fuda Cancer Hospital. Twelve female Tibet mini-pigs pigs, weighing 25–35 kg each, were provided by the Animal Experimental Center of Southern Medical University. IRE (NanoKnife; AngioDynamics, Queensbury, NY) to the colon wall was performed in a total of 24 lesions (two lesions per animal). Animals were fasted for 12 h and subjected to an enema 9 h prior to the operation in order to clean the intestinal tract.

### IRE ablation

Animals were anesthetized by intramuscular injection of ketamine (6–8 mg/kg) and general anesthesia was maintained with isoflurane (1.5–2%) by continuous inhalation. Benzene sulfonic acid cis-atracurium (0.2 mg/kg) was administered intravenously before the procedure and maintained with 10–12 μg/kg/min pump infusion (pump infusion began after intubation). The maximal dose was required to prevent severe muscle contraction during electrical pulse generation. Two 19-g single monopolar electrodes (AngioDynamics) were used in all cases. Sterile surgical techniques were used throughout the entire surgery.

**Fig 1:**
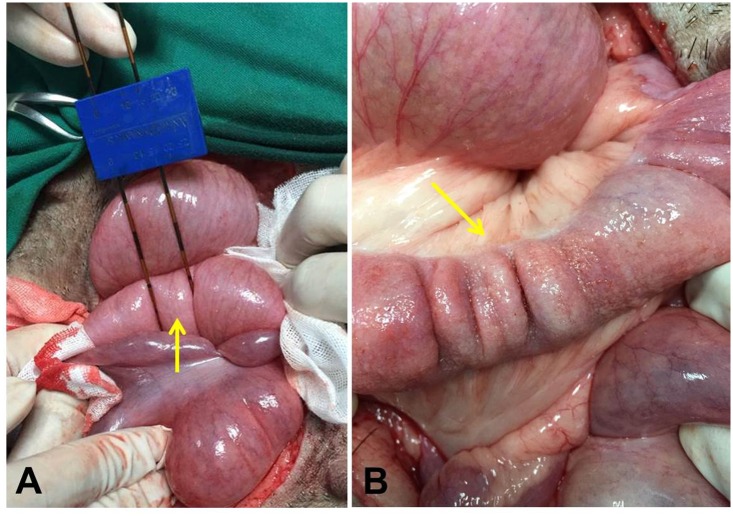
Intraoperative image of irreversible electroporation (IRE) ablation of the porcine colon. The electrodes were placed on the colon wall (arrow) with a distance between them of 1.5 cm (A). After IRE ablation the colon immediately contracted (arrow) and turned white, then red (B).

After induction of anesthesia, an 8 cm midline abdominal incision was made to expose the colon. Electrodes were gently placed on the lateral wall of the descending colon (Fig 1A). The measured distance between the two electrodes was 1.5 cm for all animals tested (with 1.5-cm exposure length). A sequence of 90 DC pulses of 1500 V/cm, 70 μs each at a frequency of 4 Hz was applied between the electrodes using a high voltage pulse generator designed for electroporation procedures (ECM 80, Harvard Apparatus). The electrical parameters used in this study are typical of those used in clinical procedures to produce irreversible electroporation without causing thermal damage to the intestinal tissue. The location of treatment was noted based on anatomy, and a suture knot was placed in the mesentery to mark the IRE treatment zone. At the end of the experiment, the abdomen wall was sutured closed, followed by the skin incision. Tissue adhesive was applied over the skin sutures.

### Postoperative management

Feeding was resumed once the pigs had recovered from surgery. Enrofloxacin (Baytril; 5 mg/kg), a fluoroquinolone antibiotic, was injected intramuscularly for 3 d postoperatively. Each animal also received meloxicam (0.4 mg/kg) orally once a day for pain, starting immediately after recovery and continuing for 3 d postoperatively. Animals were clinically monitored twice a day for signs of infection or bleeding, abdomen tenderness, incision site healing, oral intake, bowel movement, and any abnormal clinical condition. Pain was evaluated by signs of bruxation, reaction to touch, signs of discomfort when defecating, depressed demeanor, and decreased appetite. Endoscopy (EMV-3000; Shanghai OJH Medical Instrument Co., Shanghai, China) was performed 3, 14, 28 days after IRE ablation to check whether the colon had any perforation or obstruction.

### Pathological analysis

Euthanasia was performed using pentobarbital (100 mg/kg administered intravenously) 7, 14, 21 or 28 days after IRE ablation. Subsequent necropsy was performed through a midline incision, allowing an extensive exploration of the abdominal cavity. The abdominal cavity was evaluated for evidence indicative of peritonitis or perforation. The colon and adjacent muscle tissue were examined grossly, harvested immediately after death, and fixed in 10% neutral buffered formalin for pathological analysis. The lesions were color-marked before fixation using different colors for each side to ensure correct identification. The ablated colon and adjacent tissue were routinely processed, embedded in paraffin, sectioned at 4μm thickness, and stained with hematoxylin and eosin (H&E) stain or Masson’s trichrome (MT) stain. Samples of ablated colon tissue at days 7 and 28 were also stained using a terminal deoxynucleotidyl transferase dUTP nick end labeling (TUNEL) assay.

### Data collection and analysis and statistical analysis

All analyses were performed using GraphPad Prism 5 software (GraphPad Inc., San Diego, CA). Size measurement of IRE ablation zone was obtained from endoscopy findings and histopathological specimens.

## Results

### Clinical course

To investigate the effect of IRE ablation on healthy colon tissue, 12 female Tibet mini-pigs were subjected to the procedure (Fig 1A). After IRE ablation the colon contracted and turned white, then red (Fig 1B). The animals were fasted postoperatively for 12 hours and displayed poor appetite for the first two days, but this began to improve on the third day. The abdominal incisions recovered well with no signs of infection or bleeding and no obvious symptoms of discomfort. All pigs tolerated the ablation procedure without serious clinical symptoms or complications (Table 1).

**Table 1:** Clinical course following irreversible electroporation (IRE).

Subject	Perforation^†^	Obstruction^†^	Adhesion^‡^	Sacrifice time (d)
1	-	-	-	7
2	-	-	-	7
3	-	-	+	7
4	-	-	-	14
5	-	-	+	14
6	-	-	-	14
7	-	-	+	21
8	-	-	+	21
9	-	-	+	21
10	-	-	+	28
11	-	-	+	28
12	-	-	+	28

^†^ Perforation and obstruction were examined by endoscopy (days 7, 14 and 28) and by gross pathological examination postmortem.

^‡^ Fibrous adhesions on the serosal surface of the colon were identified by gross pathological examination postmortem.

### Endoscopy findings

**Fig 2:**
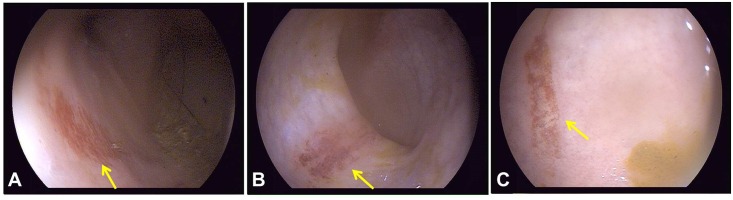
Endoscopy images after irreversible electroporation (IRE) ablation. IRE lesions were monitored by endoscopy at day 3 (**A**), day 14 (**B**) and day 28 (**C**) posttreatment, revealing the progressive shrinkage of the damaged area of colonic mucosa (indicated by arrows) and the changes in coloration over time.

**Fig 3:**
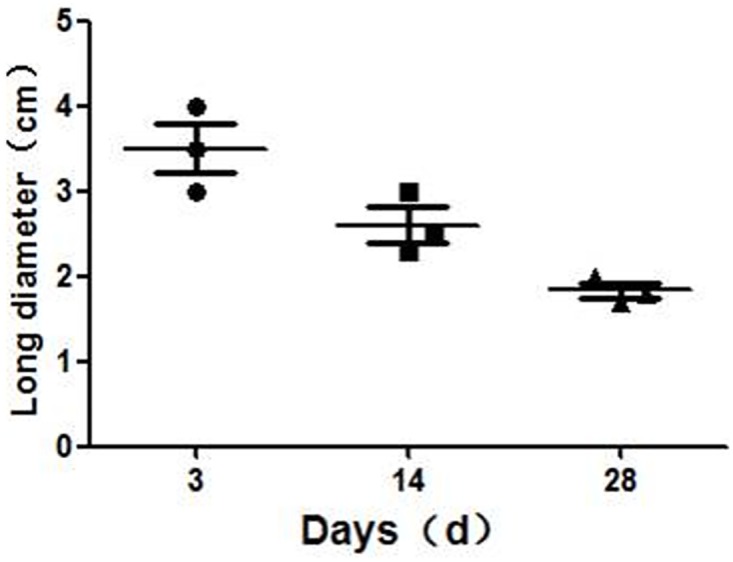
Changes in mucosal damage area determined from endoscopic measurements after irreversible electroporation (IRE) ablation.

No perforation was noted by endoscopy during follow-up of any of the animals. Endoscopic investigation three days after IRE ablation (Fig 2A) revealed a damaged area of colonic mucosa measuring approximately (3.5 ± 0.5) cm × (2.1 ± 0.2) cm, 50 cm from the anus. The mucosal surface appeared rough and showed signs of erosion and an orange-red coloration. Two weeks after IRE ablation (Fig 2B), the mucosa damage zone was smaller than before at (2.6 ± 0.4) cm × (1.3 ± 0.3) cm and was dark red. Four weeks after IRE ablation (Fig 2C), the surface of the damaged area was rough and the color remained dark red, but faded compared with that observed at 2 weeks after ablation. The mucosa damage zone was (1.8 ± 0.2) cm × (0.8 ± 0.3) cm. Over time, the area of mucosal injury gradually decreased (Fig 3).

### Gross pathology findings

**Fig 4:**
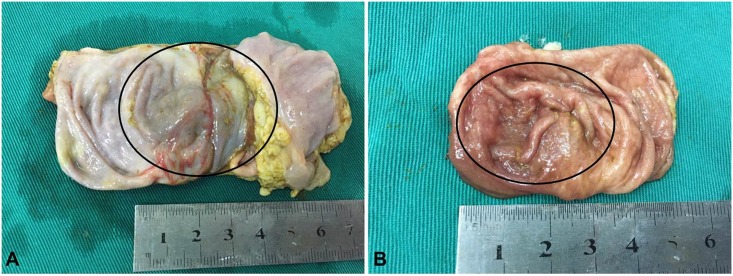
Gross pathology of the colon 28 days after irreversible electroporation (IRE) ablation. On the serosal surface (**A**), a fibrous scar was observed in the ablation area (circle). On the mucosal surface (**B**) the ablated colon appeared grossly normal.

Gross pathological examination revealed a well-demarcated focal lesion in ablated colon tissue but no perforation of the colon. Twenty-eight days after IRE ablation, there was no clear boundary between the ablation region and the surrounding normal tissue, while the ablated lesions appeared light red, a fibrous scar was observed in the serosal surface and the mucosal surface appeared similar to normal colon tissue (Fig 4).

### Pathological analysis

**Fig 5:**
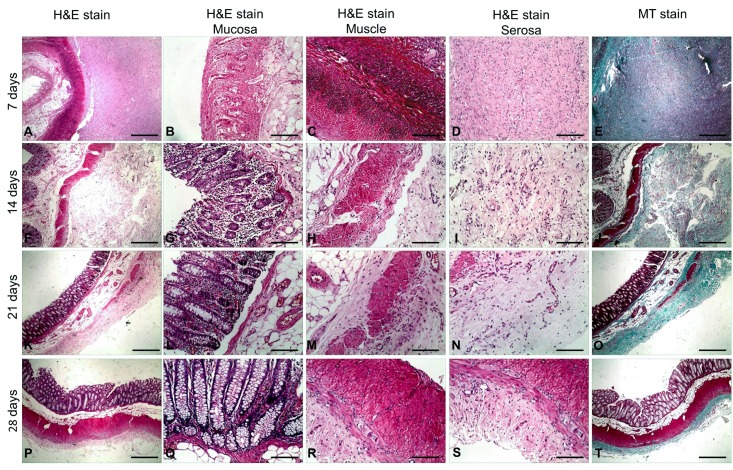
Histopathology of the colon wall after irreversible electroporation (IRE) ablation. **(A-E)** The colon wall shows necrosis in the mucosa, submucosa and muscle layers 7 days after IRE ablation and significant fiber proliferation in the serosa layer (**E**). Inflammatory cell infiltration **(C)** and significant proliferation of serosa layer fibers **(D-E)** can also be observed. **(F-J)** Fourteen days after IRE ablation, the colon wall shows hyperplasia of the mucosal glands (**G**), degeneration and necrosis of the muscular layer (**H**), thickening and fibrosis of the serosa (**I, J**). **(K-O)** Twenty-one days after IRE ablation, the muscle layer was almost absent (**K**), replaced with fibrous granulation tissue (**N**). **(P-T)** Twenty-eight days after IRE ablation, the colon wall appears histopathologically normal, with the muscle layer regenerated **(R)** and the serosa returned to normal thickness **(S, T)**. Scale bars in A, E, F, J, K, O, P and T = 2.5 mm (20× magnification). Scale bars in B–D, G–I, L–N and Q–S = 500 μm (100× magnification). Hematoxylin and eosin stain (H&E stain), Masson’s trichrome stain (MT stain).

**Fig 6:**
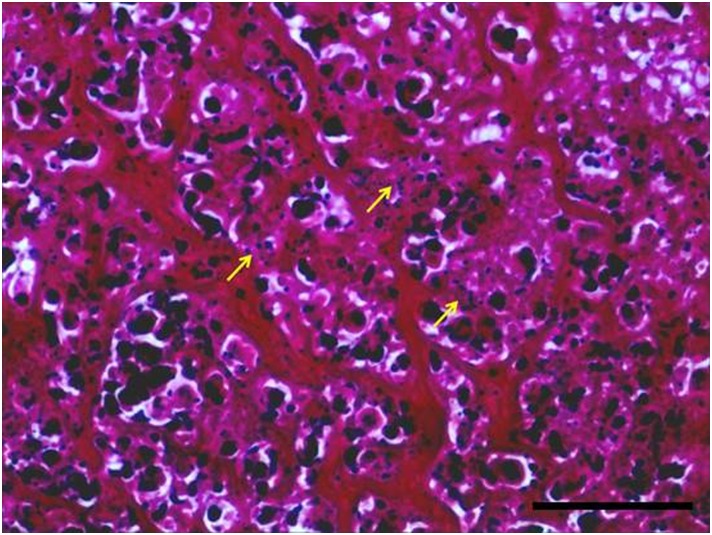
High magnification histopathology of the muscle layer 7 days after irreversible electroporation (IRE). A large number of apoptotic bodies of varying sizes (arrow) are visible in the residual necrotic muscle layer after staining with hematoxylin and eosin (H&E). Scale bar = 100 μm; 400× magnification.

Seven days after IRE treatment, histopathological examination showed necrosis of the colonic mucosa, submucosa and muscle layer and infiltration of inflammatory cells into the lesion (Fig 5A–5C). The serosa layer was thickened and significant fiber proliferation was observed (Fig 5D and 5E). A large number of apoptotic bodies were also identified in the residual muscle layer at this time point (Fig 6). Fourteen days after IRE treatment, mucosal gland hyperplasia (one of the initial signs of recovery) and fibrous tissue hyperplasia were observed in the serous membrane, while the residual muscle showed degeneration and necrosis (Fig 5F–5J). Twenty-one days after IRE ablation, we observed signs of mucosal repair, disappearance of ruptured muscle fibers and replacement with fibrous granulation tissue (Fig 5K–5O). Twenty-eight days after IRE ablation, the longitudinal and circumferential muscle layers were distinguishable and the colon layers appeared close to normal histopathologically (Fig 5P–5T). This recovery demonstrates that the colon remains structurally intact after IRE ablation and is capable of gradual recovery.

**Fig 7:**
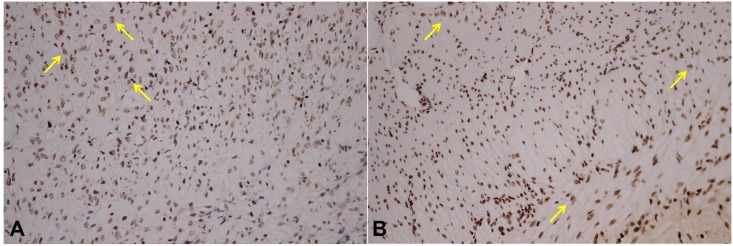
TUNEL assay for apoptotic cells after irreversible electroporation (IRE). Apoptosis detected with TUNEL (yellow arrow). Apoptotic cells in the ablation area can be visualized by brown-yellow nuclear staining; **(A)** 7 days and **(B)** 28 days after IRE ablation. 400× magnification.

**Fig 8:**
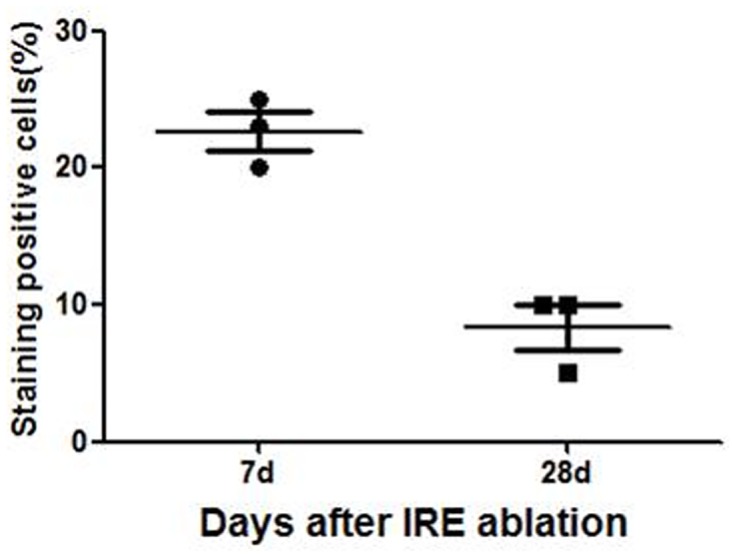
Changes of apoptotic markers in the TUNEL assay after IRE ablation.

In addition to the necrosis observed through H&E staining, the IRE-ablated zone also showed high levels of apoptotic markers in the TUNEL assay (Fig 7). The percentage of apoptotic body 7 days after IRE ablation was higher than that 28 days after ablation (Fig 8).

## Discussion

IRE uses short pulses (70–90 μs) of high voltage stimulation (maximum 3000 V) to induce cell membrane porosity which leads to slow/protracted cell death. IRE has been used clinically in the treatment of locally advanced soft tissue tumors since 2008. The ability to apply IRE in a minimally invasive manner and the reported safety of this procedure has led to a recent surge in its clinical use[15]. IRE affects only the target tissue and spares the surrounding structures, producing a clear boundary between the treated and untreated area; it does not produce a heat sink effect and can therefore by used treat tumors near large blood vessels, and it has been suggested that the use of IRE may allow rapid regeneration of healthy tissues because it selectively targets cell membranes, preserving connective tissue scaffolding[8, 16, 17]. Furthermore, since the energy supplied by IRE primarily targets cell membranes, proteins, the extracellular matrix, and critical structures such as blood vessels and nerves, all remain unaffected[17].

Previous studies using liver models showed complete sparing of bile ducts within the ablation zone[18], while similar *in vivo* dog prostate study showed sparing of the urethra, vessels, nerves, and rectum after IRE ablation[10]. In a study by Onik et al., there was a rapid resolution of the treated area when IRE was used in the treatment of prostate tumors[10]. Structures such as the urethra, larger blood vessels, nerves, and rectum, were not affected by IRE under the parameters applied in their study. Sparing the rectum by IRE would overcome the limitations of prostate ablation and would make IRE a promising ablation tool in patients with prostatic cancer or other cancers that may be adjacent to or infiltrating the rectum, such as carcinomas of the bladder, gynecological carcinomas, and recurrent carcinomas of the rectum[10]. Complementary to the study of Onik et al., Schoellnast et al. found no perforation after IRE ablation adjacent to the rectum[14]. They suggested that IRE ablation adjacent to the rectum may be uneventful if the rectum wall is mobile and free to contract. In contrast, IRE-ablation of the rectum may be harmful if the rectum wall is fixed adjacent to the IRE-probe. In this study, we placed electrodes on the colon lateral wall and used gauze to fix the colon at the distal target ablation site with freedom to move, corresponding to the study by Schoellnast et al. When IRE ablation was performed, the colon initially contracted, which may be a mechanism of self-preservation. Endoscopy analysis at different time points following IRE ablation showed that the damaged colon mucosa gradually repaired and the damaged area gradually decreased in size, indicating recovery of the colon.

A defined margin in the IRE ablated lesions could be observed in our study by both macroscopic and microscopic evaluation using H&E stain and MT stain. These well-controlled and focused ablation zones, producing complete cell death in the area of ablation, were seen in all 24 ablation zones. After IRE ablation, cellular homeostasis was preserved, as was the structural scaffolding as indicated by the recovery of the tissue. This behavior represents an important advantage of IRE compared to other therapeutic interventions, as it allows effective ablation of the tumor without damaging the underlying architecture of the colon tissue. Seven days after IRE ablation, fibrous tissue of serous layer hyperplasia significantly; after 14 days, mucosal glands appeared to show regeneration; after 21 days, the residual necrotic muscle was replaced with fibrous granulation tissue; after 28 days, the ablated colon tissues had almost recovered to baseline conditions. Our study indicated that the colon remained structurally intact after IRE ablation and was able to repair itself gradually over the study period. Although most tissues of the colon were damaged by IRE ablation, the ablated tissue could recover due to the preservation of connective tissue scaffolding, which supports our previous hypothesis.

Due to the preservation of connective tissue scaffolding and the regeneration of colon tissue after IRE ablation, this technique may feasibly be used as a treatment for tumors adjacent to or violating the colon tissue, such as pelvic tumors or abdominal wall tumors, in which any surgical ablation will inevitably lead to colon damage. It may also prove valuable for patients who have undergone resection but experience recurrence and for nonsurgical candidates with advanced disease who are not eligible for other treatment options. Furthermore, there is an increase in patient preference for minimally invasive procedures that have a lesser impact on the quality of life and less posttreatment morbidity. These considerations may make IRE a possible future candidate for minimally invasive treatment of colon tumors for palliative treatment.

There are some limitations to be considered in this study. First, the present study was conducted with the use of a short-term porcine model, which may have limited the complete progression of the lesion. A long-term study would allow the impact on normal rectum function and defecation to be assessed. Second, it is known that tumors exhibit heterogeneity that may affect electric field distribution, which may interfere with treatment. It would be essential to evaluate this technique in a tumor model before determining its usefulness for clinical application. Finally, the small sample size is another limitation of this study. Further research with a large number of cases and long-term observation is necessary to support our encouraging results.

In conclusion, our study demonstrates that IRE ablation of the colon wall can be performed to achieve complete ablation of the target area without serious treatment-related complications in a short-term porcine model. Our study suggests that IRE ablation may be a feasible treatment option for tumors located adjacent to or violating the colon wall.
